# Linguistic Disparities in Diabetes Care Quality in California Community Health Centers Before and During the COVID-19 Pandemic

**DOI:** 10.1177/21501319241229018

**Published:** 2024-02-07

**Authors:** Oscar V. Ramos, Timothy T. Brown, Hector P. Rodriguez

**Affiliations:** 1 University of California, Berkeley, Berkeley, CA, USA

**Keywords:** limited English proficiency, community health centers, COVID-19 pandemic, quality of care, diabetes, hypertension

## Abstract

**Background:**

Disparities in diabetes care quality may have increased for patients with limited English language proficiency (LEP) compared to non-LEP patients during the COVID-19 pandemic. Changes in diabetes care quality for adult LEP and non-LEP patients of community health centers (CHCs) were examined from 2019 to 2020.

**Methods:**

Adults with Type 2 diabetes (n = 15 965) of 88 CHC sites in California and with 1+ visit/year in 2019 and 2020 from OCHIN electronic health record data were included. Multivariable regression models estimated the association of LEP status and changes in diabetes care quality from 2019 to 2020, controlling for patient sociodemographic and clinical characteristics. Interaction terms (LEP × 2020) were used to estimate differential over time changes in (1) blood pressure screening, (2) blood pressure control (<140/90 mm Hg), and (3) hemoglobin A1c control (HbA1c <8%) for LEP versus non-LEP patients.

**Results:**

LEP and non-LEP patients with diabetes had comparable blood pressure screening and control in 2019 and in 2020. LEP patients were less likely than non-LEP patients to have their HbA1c under control in 2019 (OR = 0.85, 95% CI = 0.77, 0.96, *P* = .006) and 2020 (OR = 0.83, 95% CI = 0.75, 0.92, *P* = .001). There were no differential changes in HbA1c control over time for LEP and non-LEP patients.

**Discussion:**

Although LEP patients were less likely than non-LEP patients to have their HbA1c under control, CHCs maintained quality of care equally for LEP and non-LEP patients with diabetes during the early pandemic period.

## Introduction

Community health centers (CHCs) predominantly serve uninsured and Medicaid-insured populations and have grown rapidly over time as a usual source of care for newly insured populations. With CHCs delivering care to more than 30 million people in the country, they are widely considered by policymakers to be key resources to reduce racial, ethnic, and linguistic disparities in healthcare and health outcomes, as they serve 1 in 3 people living in poverty, and about 1 in 5 rural residents.^1^ Financial challenges coupled with lagging recruitment and retention of primary care and mental health providers, however, present barriers to quality improvement and innovation at CHCs; all of which may have been exacerbated during the COVID-19 pandemic.^2^

The rising prevalence and national economic burden of diabetes in the United States, coupled with the approximate 1.5 million deaths that are directly attributed to diabetes each year underscore the urgency to disseminate evidence-based prevention and management strategies.^3,4^ Adults with diabetes require routine ongoing care which involves close monitoring and medication management and are vulnerable to exacerbations and were at higher risk of COVID-19-related complications compared to patients without diabetes.^5^ Patients with limited language English proficiency (LEP) and chronic conditions often experience barriers to accessing care and with self-management. As a result, quality of care is often lower for LEP patients compared to non-LEP patients.^6^ Evidence also indicates that LEP patients experience higher rates of medical errors than non-LEP patients due to communication challenges.^7^

CHCs play an important role in ensuring language access for low-income LEP patients nationally.^8^ There is limited empirical evidence, however, about whether CHCs were able to maintain quality of care equally for LEP and non-LEP patients with diabetes of CHCs increased during the early COVID-19 pandemic period. We advance evidence by examining changes in quality disparities for LEP patients with diabetes of CHCs between 2019 and 2020. Analyzing clinical and administrative data from electronic health records (EHRs), we examine whether linguistic disparities in Hemoglobin A1c and blood pressure management increased in 2020.

## Methods

### Data Sources

EHR data were securely sourced from OCHIN, a nonprofit health care innovation center dedicated to generating knowledge and solutions that promote quality and affordable health care in CHCs. The analytic sample is a cohort of 15 965 adults with diagnosed type 2 diabetes and with ≥1 visits per year in 2019 and in 2020 from 1 of 88 California CHC sites. Supplemental eFigure summarizes analytic sample exclusions. Complete case analyses were conducted because all covariates had levels of missing under 6.4% for the analytic sample.

### Measures

The study outcome measures are diabetes quality of care indicators endorsed by multiple professional societies and quality accreditors.^9,10^ To investigate changes in diabetes care quality between 2019 and 2020, we assessed (1) blood pressure testing (if a patient received an annual test or not), (2) HbA1c control (<8%), and (3) blood pressure control (<140/90 mm Hg). Almost all patients had HbA1c testing in 2019 (99.91%) and 2020 (99.96%). We did not assess HbA1c testing as an outcome measure due to limited variation over time. We still assess HbA1c control as an outcome measure, however, because it varied over time and is an important indicator of diabetes care management.

### Main Independent Variable

Patients were LEP, or have limited English language proficiency, if their preferred language for medical appointments was any language other than English. Patients with English as their preferred language were considered non-LEP.

### Control Variables

We controlled for patient age, race/ethnicity, sex, federal poverty level percentage, body-mass index (BMI), clinical morbidities, and insurance type in our regression models based on past studies examining correlates of diabetes care quality.^11,12^ A count measure of 13 comorbidities was calculated and included as a covariate in regression analyses. The 13 clinical comorbidities often co-occur with diabetes and included hypertension, congestive heart failure, cardiovascular disease, congenital heart disease, diabetic retinopathy, secondary diabetes, mobility impairment, substance abuse, alcohol use, tobacco use, depression, anxiety/post-traumatic stress disorder, and other mental health conditions.

### Statistical Analyses

Multivariable logistic regression models were used to estimate the association of LEP status, year (2019 vs 2020), and diabetes quality of care (blood pressure testing measure and blood pressure and HbA1c control measures). Multivariable linear regression models estimated the association of LEP status and diabetes quality of care (HbA1c and blood pressure values). Logistic and linear regression models controlled for patient sociodemographic characteristics and clinical comorbidities. An interaction term (LEP × 2020) was estimated in each model to assess differential changes in study outcomes by LEP status. Robust standard errors were used to account for patient clustering within CHC sites. Adjusted levels were estimated using the final regression model for each study outcome, holding all covariates at their means. Statistical analyses were performed using Stata software (version 17.0; SE-Standard Edition; StataCorp LLC).

### Sensitivity Analysis

To assess the robustness of our main results to different outcome measure specifications, we (1) used HbA1c and blood pressure values as continuous measures using multivariable linear regression, controlling for the same covariates as the binary models, and (2) redefined the cut points for blood pressure (<130/80 mm Hg) and HbA1c (<9%) control in the binary regression models, controlling for the same covariates. We chose these alternative cut points based on definitions from the American Diabetes Association (ADA) and Healthcare Effectiveness Data and Information Set (HEDIS) specifications.^9,13^

## Results

### Descriptive Analyses

The analytic sample was predominantly female (57.7%) and of Hispanic/Latinx ethnicity (62.5%) (Table 1). The mean age was 57 years with a standard deviation (SD) of 12 years. Most patients were LEP (57.3%) with Spanish (53.9%), English (42.6%), and Tagalog (0.61%) being the most common preferred languages. The mean BMI is 32.2 (SD = 7.2), which indicates high obesity prevalence.

**Table 1.** Summary of Patient Characteristics (2019), by English Language Proficiency.

	Overall	Patients with limited English proficiency	Patients with English language proficiency
Patient n	15,965 (100)	9,154 (57.3)	6,811 (42.7)
Language preference (n, %)			
Spanish	8632 (53.9)	8632 (100)	0 (0)
English	6811 (42.6)	0 (0)	6811 (100)
Tagalog	97 (0.61)	97 (100)	0 (0)
Vietnamese	53 (0.33)	53 (100)	0 (0)
Hmong	40 (0.25)	40 (100)	0 (0)
Mandarin	39 (0.24)	39 (100)	0 (0)
Age (mean (SD))	57 (11.6)	56.9 (11.1)	57.2 (12.1)
Race/ethnicity (n, %)			
Hispanic/Latinx	9971(62.5)	8397 (84.2)	1574 (15.8)
White	3513 (22)	192 (5.5)	3321 (94.5)
Asian	1146 (7.2)	343 (29.9)	803 (70.1)
Black /African American	607 (3.8)	8 (1.3)	599 (98.7)
Other	495 (3.1)	174 (35.2)	321 (64.8)
AIAN or NHPI	233 (1.5)	40 (17.2)	193 (82.8)
Female (%)	9217 (57.7)	5745 (62.3)	3472 (37.7)
Federal poverty level^a^ (mean (SD))	80.2 (108.5)	62.3 (72.8)	105.8 (141.1)
Homeless (n, %)	56 (0.4)	36 (64.3)	20 (35.7)
Insurance status (n, %)			
Medicaid	5347 (33.5)	2901(54.3)	2446 (45.7)
Medicare	4566 (28.6)	1986 (43.5)	2580 (56.5)
Uninsured	2782 (17.4)	1886 (67.8)	896 (32.2)
Other public	1898 (11.9)	1730 (91.2)	168 (8.8)
Private	1372 (8.6)	651 (47.5)	721 (52.5)
Body Mass Index (mean SD))	32.2 (7.2)	31.4 (6.3)	33.1 (8.2)
Comorbidities (mean (SD))	1.7 (1.4)	1.5 (1.2)	2.1 (1.5)
HbAlc value (mean (SD))	7.8 (1.9)	8.0 (1.9)	7.6 (1.8)
HbA1c testing (n, %)	15951 (99.9)	9147 (57.3)	6804 (42.6)
HbA1c control (n, %)	10 274 (64.4)	5541(53.9)	4733 (46.1)
Systolic blood pressure value (mean (SD))	131.3 (18.6)	130.4 (19.0)	132.2 (18.2)
Diastolic blood pressure value (mean (SD))	75.9 (9.9)	74.6 (9.3)	77.4 (10.3)
Blood pressure testing (n, %)	11 485 (71.9)	6133 (53.4)	5352 (46.6)
Blood pressure control (n, %)	7907 (49.5)	4360 (55.1)	3547 (44.9)

Abbreviations: AIAN, American Indian or Alaskan Native; NHPI; Native Hawaiian or Other Pacific Islander.

a ‘Federal Poverty Level’ is a percentage with a range from 0 to 1400.

LEP adult patients with diabetes had higher mean HbA1c values compared to non-LEP adult patients with diabetes in 2019 (7.9% vs 7.6%) and in 2020 (8.1% vs 7.6%). Decreases in blood pressure testing were observed between 2019 and 2020 for both LEP (67%-48.2% tested) and non-LEP patients (78.6%-55.7% tested). LEP adult patients with diabetes had lower systolic and diastolic mean values in 2019 (130.4/74.6 mm Hg) and in 2020 (131.9 / 75.2 mm Hg), compared to non-LEP patients in 2019 (132.2 / 77.4 mm Hg) and in 2020 (133.4 / 77.7 mm Hg).

### Adjusted Analyses

In multivariable regression analyses, LEP and non-LEP patients with diabetes had comparable blood pressure testing in 2019 and in 2020 (Table 1). LEP patients had slightly smaller decrements in blood pressure testing over time compared to non-LEP patients (OR = 1.31, 95% CI = 0.99, 1.72, *P* = .058; Tables 1 and 2).

**Table 2.** Logistic Regression Results: Association of Limited English Proficiency and Changes in Diabetes Quality of Care from 2019 to 2020.

Study outcome	Model 1 (2019 analyses)	Model 2 (over time analyses)		
	LEP vs non-LEP	LEP vs non-LEP	2020 year	LEP*2020 Year
HbA1c control (<8%)	0.85 (0.77, 0.96)**	0.83 (0.75, 0.92)**	0.96 (0.91, 1.02)	0.95 (0.89, 1.02)
Blood pressure testing	1.13 (0.84, 1.51)	0.92 (0.68, 1.25)	0.34 (0.25, 0.47)***	1.31 (0.99, 1.72)
Blood pressure control (<140/90 mmhg)	1.10 (0.98, 1.28)	1.10 (0.97, 1.24)	.93 (0.84, 1.02)	0.96 (0.85, 1.09)

Abbreviation: LEP, limited English proficiency.

Results are adjusted odds ratios with 95% confidence intervals. Model is adjusted for patients’ age, race/ethnicity, sex, BMI, federal poverty level percentage, insurance, and the comorbidity count measure for all 13 comorbidities. Robust standard errors accounted for patients clustering within CHC sites.

* *P* < .05. **P < .01. *** < .001.

LEP and non-LEP patients had comparable levels of blood pressure control (<140/90 mm Hg) in 2019 (OR = 1.10, 95% CI = 0.98,1.28, *P* = .093) and in 2020 (OR = 1.10, 95% CI = 0.97, 1.24, *P* = 0.127). There were no differential changes in blood pressure control between LEP and non-LEP patients over time.

LEP patients were less likely to have their HbA1c under control (<8%) in 2019 (OR = 0.85, 95% CI = 0.77, 0.96, *P* = .006) and in 2020 (OR = 0.83, 95% CI = 0.75, 0.92, *P* = .001) compared to non-LEP patients. There were no statistically significant differences in decreased HbA1c control for LEP patients compared to non-LEP patients.

**Figure 1.**
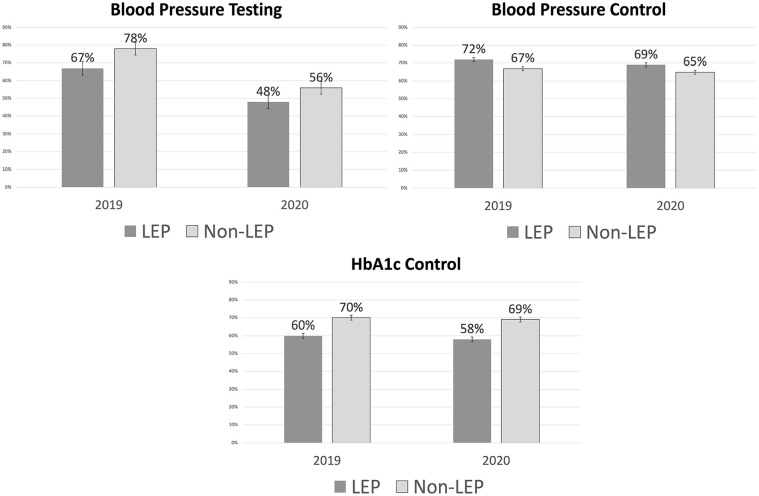
Adjusted study outcomes, by limited English proficiency status and year. Blood pressure testing was defined as whether a patient received an annual test or not). Blood pressure control was defined as <140/90 mm Hg. HbA1c control was defined as <8%.

Adjusted levels for each of the study outcomes, stratified by LEP status over time (2019 vs 2020), are presented in the Figure 1 to illustrate the regression results.

### Sensitivity Analyses

Linear regression model results with continuous outcome measures for HbA1c and blood pressure (Supplemental eTable 1) were consistent with the binary specifications of the outcome measures. When HbA1c control was defined as <9% instead of <8%, the results were also consistent (Supplemental eTable 2). In contrast to the main results, when blood pressure control was defined as <130/80 mm Hg instead of <140/90 mm Hg, LEP patients with diabetes were slightly *more* likely to have their blood pressure under control in 2019 (OR = 1.20, 95% CI = 1.06, 1.36, *P* = .004) and in 2020 (OR = 1.16, 95% CI = 1.03, 1.32, *P* = .017). However, there were no statistically significant differences in blood pressure control (<130/80 mm Hg) changes between LEP and non-LEP patients over time.

## Discussion

This study, the first to assess quality of care for adult LEP patients of CHCs with diabetes before and during the COVID-19 pandemic, found that LEP patients were less likely than non-LEP patients to have their HbA1c under control. This pattern is consistent with evidence indicating that Latinos with diabetes had less consistent HbA1c monitoring and had higher mean HbA1c values compared to White patients.^14,15^ Contrary to our expectations, our main analyses indicate that LEP and non-LEP patients had comparable blood pressure testing and control in 2019 and in 2020. We found that upon being tested, LEP patients were more likely to have control of their blood pressure (71.2%) compared to non-LEP patients (66.3%) (Figure 1), contrary to past evidence indicates that LEP patients have higher odds of out-of-range blood pressure compared to non-LEP patients.^16^ These findings were consistent when we used an alternative cutpoint (<130/80 mm Hg) to define blood pressure control, underscoring the robustness of the main results. Taken together, our results indicate that (1) CHCs were equally, if not more, successful at maintaining blood pressure testing for LEP patients with diabetes during the early pandemic, and (2) once tested, LEP patients had better blood pressure control. CHCs in our sample may have engaged in targeted outreach to LEP populations during the pandemic and were able to provide culturally competent care.^17,18^ Additional evidence is needed to help CHCs remove barriers to blood pressure management among LEP patients with diabetes.^19^ In recent years, medical experts have shifted their focus to systolic pressure in adults aged 50 years and older as a better way to predict future cardiovascular events and death and emphasized the importance of measuring at-home blood pressure to identify white-coat hypertension to increase earlier, more accurate diagnoses.^20-22^ At-home blood pressure measurement and image-based instructions for self-monitoring may facilitate hypertension management for adults with diabetes of CHCs.^23^

Importantly, we found that quality of care declined for adults with diabetes at CHCs during the early pandemic period (2020), irrespective of LEP status. These results are consistent with other studies documenting declines in routine HbA1c monitoring and control during the early pandemic period.^3^ Our study found evidence that disparities in diabetes care quality for LEP and non-LEP patients of CHCs were consistent over time., which suggests that CHCs were equally attentive to their LEP versus non-LEP patients in terms of care management during the pandemic or made extra efforts to maintain quality for LEP patients. In fact, LEP patients had smaller decrements to blood pressure testing during the pandemic compared to non-LEP patients. This may also suggest CHCs were able to maintain ongoing diabetes care management and successfully conduct outreach in multiple languages to reach these LEP target populations.^24^ The results highlight the impact of CHCs’ commitment and competencies in serving linguistically diverse populations during the COVID-19 pandemic.^22^

There are some limitations that should be considered when interpreting our findings. First, our results may not generalize to patients with less visit per year given our analytic sample restrictions, which were needed to examine changes in quality of care. Second, although we examined a patient cohort over time, we are not able to disentangle COVID-19 pandemic specific effects from other simultaneous policies. Third, we assessed annual quality of care measures, which means that patients who received testing between January and March 13, 2020 may have met quality indicators for 2020 before the national emergency period. Fourth, information about interpreter services or clinician-patient language concordance were not available and inclusion of these variables could alter the results. Finally, no information is available about other important quality measures, including medication adherence, dilated eye examinations to assess diabetic retinopathy, medical attention for nephropathy, or patient-reported outcomes. Assessing these outcomes will be an important next step for future research examining differences in quality of care between LEP and non-LEP patients with diabetes.

## Conclusion

Although quality of diabetes care declined in 2020, CHCs were able to maintain quality equally for adult LEP and non-LEP patients with diabetes amid a major economic and public health shock that disproportionately impacted low-income populations. To our knowledge, our study is the first to provide empirical evidence that CHCs maintained quality of diabetes care equally for patients with limited English proficiency and English-speaking patients during the early pandemic period. To reduce inequities in diabetes care quality for LEP patients, generating and disseminating practice-based evidence for managing language access for chronic care management for CHC patients should be a high policy priority.

## Supplemental Material

sj-docx-1-jpc-10.1177_21501319241229018 – Supplemental material for Linguistic Disparities in Diabetes Care Quality in California Community Health Centers Before and During the COVID-19 Pandemic Click here for additional data file. Supplemental material, sj-docx-1-jpc-10.1177_21501319241229018 for Linguistic Disparities in Diabetes Care Quality in California Community Health Centers Before and During the COVID-19 Pandemic by Oscar V. Ramos, Timothy T. Brown and Hector P. Rodriguez in Journal of Primary Care & Community Health
