# Supplementary material for: Linguistic Disparities in Diabetes Care Quality in California Community Health Centers Before and During the COVID-19 Pandemic
Source: J Prim Care Community Health. 2024 Feb 7;15:21501319241229018. doi: 10.1177/21501319241229018 (PMC10851749; doi:10.1177/21501319241229018)
Supplement: sj-docx-1-jpc-10.1177_21501319241229018 – Supplemental material for Linguistic Disparities in Diabetes Care Quality in California Community Health Centers Before and During the COVID-19 Pandemic [file sj-docx-1-jpc-10.1177_21501319241229018.docx]

**Linguistic Disparities in Diabetes Care Quality in California Community Health Centers Before and During the COVID-19 Pandemic**

Supplementary Information

*eFigure*. Inclusion/Exclusion Flow Diagram for Original Analytic Sample


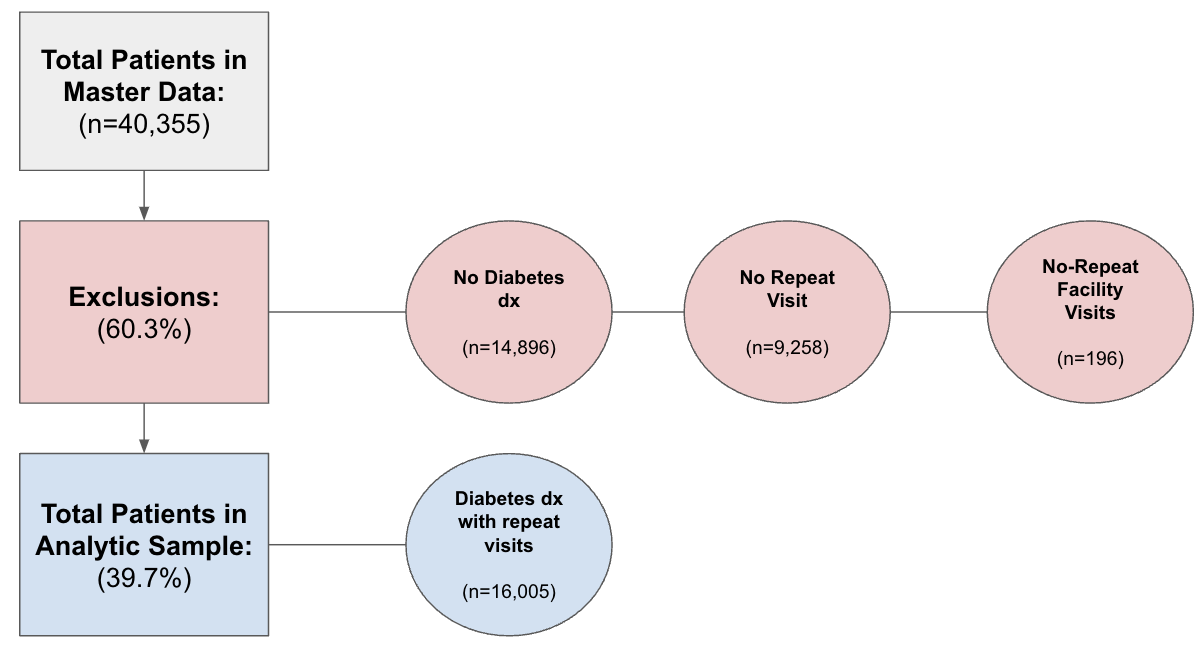


*eTable 1.* Sensitivity Analysis: Results of Adjusted Multivariable Linear Regression Models between LEP Status and Continuous Diabetes Quality of Care Outcomes in 2019 and 2020

|  |  | **A1c Value** | | | **Systolic Blood Pressure Value** | | | **Diastolic Blood Pressure Value** | | |
| --- | --- | --- | --- | --- | --- | --- | --- | --- | --- | --- |
|  |  | **𝛃** | **p** | **95% CI** | **𝛃** | **p** | **95% CI** | **𝛃** | **p** | **95% CI** |
| **2019** | **LEP** | 0.16 | 0.006 | (0.05, 0.27) | -0.67 | 0.368 | (-2.15, 0.81) | -1.63 | 0.000 | (-2.39, -0.88) |
| **2020** | **LEP** | 0.17 | 0.002 | (0.07, 0.28) | -0.51 | 0.449 | (-1.84, 0.82) | -1.53 | 0.000 | (-2.28, -0.77) |
|  | **POST** | 0.05 | 0.012 | (0.01, 0.10) | 1.11 | 0.009 | (0.29, 1.93) | 0.27 | 0.232 | (-0.17, 0.70) |
|  | **LEP* POST** | 0.03 | 0.373 | (-0.03, 0.09) | 0.54 | 0.320 | (-0.53, 1.61) | 0.32 | 0.163 | (-0.13, 0.77) |
| LEP= limited English proficiency; Results are adjusted odds ratios with 95% confidence intervals. Model is adjusted for patients’ age, race/ethnicity, sex, BMI, federal poverty level percentage, insurance, and the comorbidity count measure for all 13 comorbidities. Robust standard errors accounted for patients clustering within CHC sites. | | | | | | | | | | |

*eTable 2.* Sensitivity Analysis: Results of Adjusted Multivariable Logistic Regression Models between LEP Status and Binary Diabetes Quality of Care Outcomes in 2019 and 2020 using ADA and HEDIS Specifications

|  |  | **A1c Control**  **(<9%)** | | | **Blood Pressure Control**  **(<130/80 mmhg)** | | |
| --- | --- | --- | --- | --- | --- | --- | --- |
|  |  | **OR** | **p** | **95% CI** | **OR** | **p** | **95% CI** |
| **2019** | **LEP** | 0.83 | 0.005 | (0.73, 0.95) | 1.20 | 0.004 | (1.06, 1.36) |
| **2020** | **LEP** | 0.81 | 0.001 | (0.71, 0.91) | 1.16 | 0.017 | (1.03, 1.32) |
|  | **POST** | 0.92 | 0.033 | (0.85, 0.99) | 0.88 | 0.003 | (0.81, 0.96) |
|  | **LEP*POST** | 1.01 | 0.749 | (0.93, 1.11) | 0.98 | 0.709 | (0.87, 1.10) |
| Note: LEP= limited English proficiency; Results are adjusted odds ratios with 95% confidence intervals. Model is adjusted for patients’ age, race/ethnicity, sex, BMI, federal poverty level percentage, insurance, and the comorbidity count measure for all 13 comorbidities. Robust standard errors accounted for patients clustering within CHC sites. | | | | | | | |
